# A Brief History of Nutritional Rickets

**DOI:** 10.3389/fendo.2019.00795

**Published:** 2019-11-14

**Authors:** Benjamin J. Wheeler, Anne Marie E. Snoddy, Craig Munns, Peter Simm, Aris Siafarikas, Craig Jefferies

**Affiliations:** ^1^Department of Women's and Children's Health, Dunedin School of Medicine, University of Otago, Dunedin, New Zealand; ^2^Paediatric Department, Southern District Health Board, Dunedin, New Zealand; ^3^Department of Anatomy, University of Otago, Dunedin, New Zealand; ^4^Institute of Endocrinology & Diabetes, Children's Hospital Westmead, Sydney, NSW, Australia; ^5^Discipline of Child & Adolescent Health, University of Sydney, Sydney, NSW, Australia; ^6^Department of Endocrinology and Diabetes, Royal Children's Hospital, Melbourne, VIC, Australia; ^7^Murdoch Children's Research Institute, Melbourne, VIC, Australia; ^8^Department of Paediatrics, University of Melbourne, Melbourne, VIC, Australia; ^9^Department of Endocrinology and Diabetes, Perth Children's Hospital, Perth, WA, Australia; ^10^Division of Paediatrics, Medical School, University of Western, Perth, WA, Australia; ^11^Institute for Health Research, University of Notre Dame, Freemantle, WA, Australia; ^12^Exercise Medicine Research Institute, Edith Cowan University, Joondalup, WA, Australia; ^13^Starship Children's Health, Auckland, New Zealand

**Keywords:** rickets, vitamin D, osteomalacia and rickets' diseases and disorders of/related to bone, history, public health, rickets prevention and control

## Abstract

Since first described almost a century ago, vitamin D preparations have been successfully used as a public health intervention to prevent nutritional rickets. In this manuscript, we document the periods in history when nutritional rickets was described, examine early efforts to understand its etiology and the steps taken to treat and prevent it. We will also highlight that despite the wealth of historical data and multiple preventative strategies, nutritional rickets remains a significant public health disorder. Nutritional rickets has both skeletal and extraskeletal manifestations. While the skeletal manifestations are the most recognized features, it is the extraskeletal complications, hypocalcaemic seizure and cardiomyopathy that are the most devastating features and result in reported fatalities. Reviewing this history provides an opportunity to further promote recent global consensus recommendations for the prevention and management of nutritional rickets, as well as gain a greater understanding of the well-known public health measures that can be used to manage this entirely preventable disease.

## Introduction

An adequate serum concentration of Vitamin D (acting via its active metabolite) is required for optimal absorption of calcium from the gastrointestinal tract. This vitamin D concentration or status is maintained by either endogenous production following ultraviolet B (UVB) irradiation of the skin, or from dietary intake (although without supplementation/fortification diet alone is unlikely to meet normal daily requirements). When Vitamin D deficiency occurs, calcium absorption is reduced and furthermore when accompanied by low dietary calcium intake can lead to total body calcium deficiency and compensatory hyperparathyroidism. Elevated parathyroid hormone concentrations results in phosphaturia and low serum phosphate levels with resultant abnormal bone mineralisation. In adults, this mineralisation defect results in osteomalacia (impaired mineralisation of bone matrix) and osteoporosis (reduction in the amount of bone). In children, osteomalacia can be accompanied by growth plate abnormalities (nutritional rickets). Rickets is specific to children due to their open growth plates and results from a combination of poor mineralisation of the primary and secondary spongiosa and the lack of chondrocyte terminal differentiation caused by hypophosphataemia (1). There are multiple causes of rickets, but vitamin D deficiency, usually in concert with dietary calcium deficiency, is the leading cause with an incidence of between 3 and 10.5/100,000 in prospective surveillance studies (2–5). The classic features of rickets include: flared wrists, lower limb bowing, rachitic or rickety rosary, poor growth, delayed dental eruption and enamel defects, bone pain, myopathy, and motor developmental delay (2, 4). In rare instances pathological fracture may also occur (2, 4). The most clinically significant associated feature of nutritional rickets is biochemical hypocalcaemia, which results from total body calcium depletion, and is more likely to present in infancy/early childhood (6) (i.e., periods of rapid growth). As extracellular calcium is essential for normal nerve and muscle function, low serum calcium can result in neuromuscular excitability which in severe cases can result in tetany and hypocalcaemic convulsions (4). Hypocalcaemic myopathy can also manifest in cardiomyopathy and occasional death.

Nutritional rickets can be almost fully prevented by universal supplementation with 400 IU of cholecalciferol (vitamin D) of all infants for the first 12 months of life and the continued supplementation of all children at risk of vitamin D deficiency (4). In this review we provide a historical perspective on nutritional rickets. This perspective can be used to understand ongoing challenges in prevention, and highlights that rickets is not a new or modern disease. It also provides a further opportunity to promote recent global consensus recommendations on prevention and management of nutritional rickets (4).

## Historical Perspective

**Figure 1:**
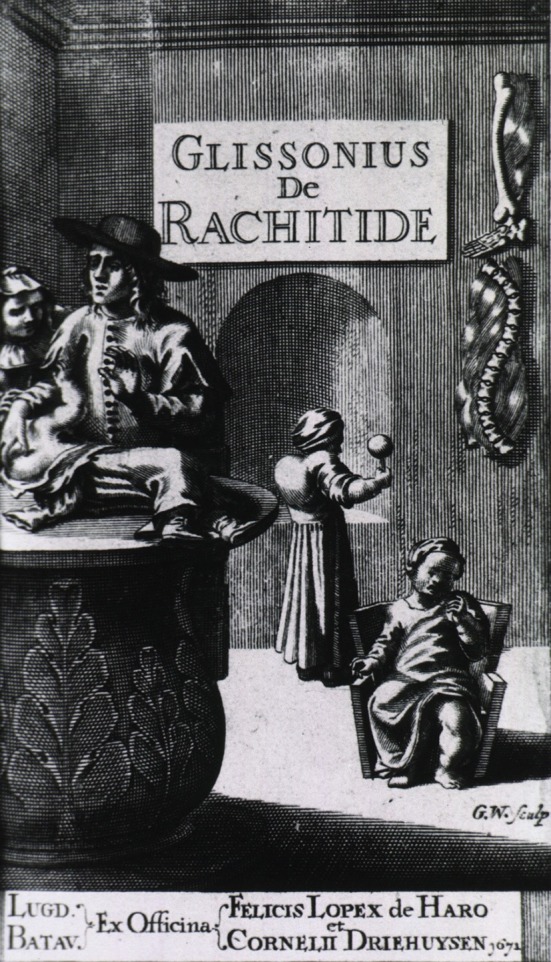
Glissonius de Rachitide—“Glisson examines child with rickets as the mother looks on. Two more children with rickets play in the background and bones deformed by rickets hang on the wall.” The US National Library of Medicine digital collection http://resource.nlm.nih.gov/101434430 (open access).

The origins of the term “rickets” remains somewhat unclear. However, it likely originates from the German word “wricken” which translates to “twisted” (7). The first clear descriptions of rickets occurred in the seventeenth century, by the English physicians Daniel Whistler (1645), and Francis Glisson (1650) (8) (see Figure 1). However, early descriptions were recognized well before this time. In hindsight, not only was presumed rickets described in early Roman and Greek and medical writings of the first and second century AD (9), but the essential need for vitamin D is considered to be a leading theory to explain the evolution from dark to light of skin color in humans, in regions of seasonal variation in UVB availability (10). Rickets is also increasingly documented in archaeological evidence from pre-industrial Europe. Skeletal changes indicative of childhood rickets and/or adult osteomalacia have been found in the archaeological records of fourth century France (11), sixteenth century Italy (12), Roman Dorset, UK (13), and Medieval North Yorkshire, UK (14).

However, the number of reported paleopathological cases of rickets increases significantly from the seventeenth to nineteenth centuries, coinciding with the industrial revolution (the “first wave of rickets”). This suggests that overcrowding, an increase in specialized indoor occupations, and poor air quality (i.e., smog), and possibly decreased calcium in the diet [via an increasing dietary role of bread at the expense of diary (15)] all contributed to vitamin D deficiency and nutritional rickets as a consequence of decreased calcium and UVB exposure (16). Although both urban and rural communities were affected (17), the prevalence of rickets was higher in urban centers during this period, and the condition appears to be more common in lower socioeconomic status communities (18). However, the privileged were certainly not exempt (19).

Importantly, despite detailed scientific descriptions of rickets, its pathophysiology remained poorly understood (20, 21). A variety of causes of rickets were proposed by leading pathologists in the nineteenth century, including congenital syphilis and paternal tuberculosis infection (21). Undoubtedly these conditions were frequently comorbid, particularly in poor, industrial communities, so the confusion is understandable. A prime (but extreme) example of this lack of understanding of the pathophysiology of rickets comes from Glisson, one of the pioneers in early descriptions of rickets, who suggested various treatment options to help straighten out crooked bones, including: cautery, splinting, and even pendulous suspension (9).

A true modern understanding of the pathophysiological basis of rickets did not begin until the early twentieth century. In 1919, Mellanby discovered and published on the “4th vitamin,” stating “Rickets is a deficiency disease which develops in consequence of the absence of some accessory food factor or factors. It therefore seems probable that the cause of rickets is a diminished intake of an anti-rachitic factor, which is either fat-soluble factor A, or has a similar distribution to it” (22). McCollum, who had previously raised the possibility of the existence of “fat-soluble A,” subsequently named this factor “vitamin D” (as vitamins A, B, and C were already named) (23). Following these discoveries, Alfred Hess (and others), a pediatrician and nutrition researcher, pioneered cod liver oil supplementation (now known to be a rich source of vitamin D), in an African American community in 1917 (24). Hess also documented an increased risk of rickets with unsupplemented breastfeeding and changes in season (24–26), risk factors that have now been confirmed in multiple recent studies and included in recent global consensus guidelines (2, 4, 27). Also at this time, animal experiments by Shipley and Park (28) highlighted the healing of rickets with cod liver oil and UVB light exposure.

However, even with the above increased understanding, and availability of dietary supplements like cod liver oil, in early-mid twentieth century rickets continued to be common. This is beautifully illustrated in a publication originating from the Renwick hospital for sick infants in Sydney, Australia in 1929 (29). In this study, of 218 consecutive infants reviewed in their outpatient department, rickets was identified in 52%. The author also offered (mostly) perceptive advice on the prevention of rickets, some of which remain applicable today: “(1) public education in the dangers to the child of an ill-balanced diet in the mothers as regards vitamin content during pregnancy and lactation ….; (2) a stronger insistence on daily exposure of the half-naked child to the direct rays of the morning sun; (3) The routine provision to all outpatients of a stronger preparation of cod-liver oil ….” Interestingly, reported rates of rickets out of Great Britain during World War 2 are lower at 2–12% depending on location (15).

**Figure 2:**
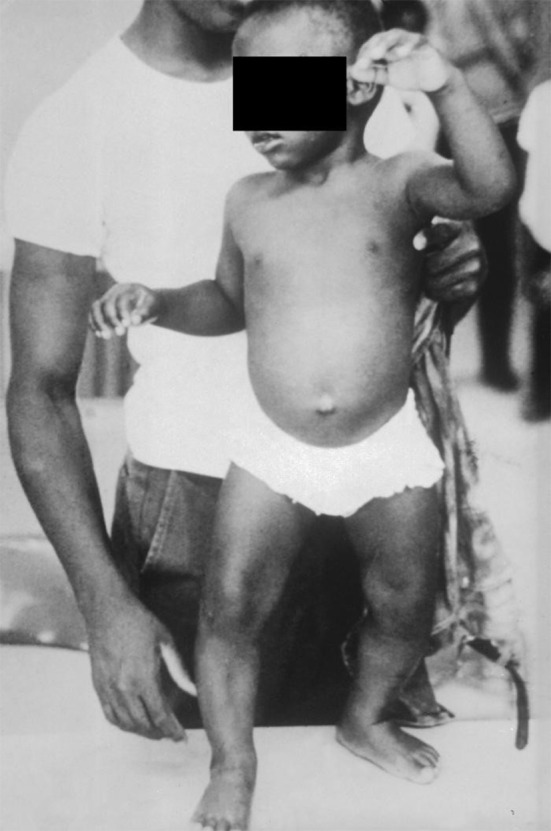
Child suffering from vitamin D deficiency rickets in 1970—image obtained from the Center for Disease Control via the Public Health Image Library (PHIL) at https://phil.cdc.gov/phil/details.asp (open access).

Food fortification, dietary supplementation with cod liver oil, and increased use of vitamin D fortified infant formula led to a decrease in rickets over subsequent decades (30). This was until increasing immigration in the 1960's and 70's of dark-skinned individuals from locations such as the West Indies, India, and South Asia to England and Europe led to the identification of a “second wave” of rickets (31) (see Figure 2). Again, a public health campaign of vitamin D supplementation targeting these recent immigrants predisposed to vitamin D deficiency reduced rickets presentations (32).

Unfortunately, once again complacency has set in, and we are now in the middle of a “third wave” of rickets. This “third wave” has been documented in prospective surveillance studies of vitamin D deficiency rickets in Australia (4), Canada (4), and New Zealand (1), as well as in multiple retrospective studies from across the globe. In part, this is caused by reduced UVB exposure due to sun avoidance measures (such as sunscreen and clothing, including for cultural reasons) and other modern lifestyle factors. Complacency and our short memories for diseases from the past, including clear lapses in awareness of preexisting public health strategies for vitamin D supplementation to high risk populations have also contributed. These strategies are well-covered in recent global consensus guidelines for prevention and management of vitamin D deficiency rickets, with recommended treatment and prevention doses of vitamin D for nutritional rickets in children presented in Table 1 (4).

**Table 1:** Suggested vitamin D (cholecalciferol) doses for the prevention and treatment of nutritional rickets in children (4).

**Age**	**Daily rickets treatment dose for 90 days, IU**	**Single treatment dose, IU**	**Maintenance/prevention daily dose, IU**
<3 months	2,000	NA	400
3-12 months	2,000	50,000	400
>12 months to 12 years	3-6,000	150,000	600
>12 years	6,000	300,000	600

*NA, Not available. Reassess response to treatment after 3 months as further treatment may be required. Ensure a daily calcium intake of at least 500 mg. For conversion from IU to μg, divide by 40*.

In conclusion, rickets is not just a modern disease. However, we can learn from the past, and an awareness of successful vitamin D supplementation campaigns over the last century is helpful. If we are to successfully prevent nutritional rickets in children, efforts to promote the existing clear guidelines on prevention are needed (4). The important take home messages include: ongoing efforts to promote existing food fortification and vitamin D supplementation policy, with a focus on targeting children aged <3 years of age, particularly those with any of the following risk factors: dark skin (especially those of African and South Asian decent), exclusive breastfeeding, and residing at higher latitudes. Please read the global consensus recommendations (4) to help in the worldwide effort to eradicate this rare but fully preventable disorder.

## Author Contributions

BW conceived and wrote the first draft of the manuscript. All other authors contributed to the writing and revision of the manuscript, read, and approved the final submitted version.

### Conflict of Interest

The authors declare that the research was conducted in the absence of any commercial or financial relationships that could be construed as a potential conflict of interest.
